# Mapping seasonal human mobility across Africa using mobile phone location history and geospatial data

**DOI:** 10.21203/rs.3.rs-5743829/v1

**Published:** 2025-01-23

**Authors:** Hal E. Voepel, Shengjie Lai, Jessica Steele, Alexander Cunningham, Grant Rogers, Corrine Ruktanonchai, Nick Ruktanonchai, C Utazi, Alessandro Sorichetta, Andrew Tatem

**Affiliations:** University of Southampton; University of Southampton; University of Southampton; University of Southampton; University of Southampton; Virginia Tech; Virginia Tech; University of Southampton; University of Milan; University of Southampton

## Abstract

Seasonal human mobility data are essential for understanding socioeconomic and environmental dynamics, yet much of Africa lacks comprehensive mobility datasets. Human movement, shaped by economic needs, family responsibilities, seasonal climatic variations, and displacements, is poorly documented in many regions due to limitations of traditional methods like censuses and surveys. This study addresses these gaps by leveraging the Google Aggregated Mobility Research Dataset (GAMRD) and a Bayesian spatiotemporal framework to estimate pre-pandemic monthly mobility flows at both national and regional scales across Africa for 2018–2019. We analysed 25 countries with complete GAMRD data and developed regional models to estimate mobility in 28 additional countries with sparse or missing records, filling critical data gaps. Key predictors, including GDP per capita, underweight children, infant mortality, environmental variables like stream runoff and evapotranspiration, and covariate interactions, revealed the complexity of mobility drivers. This approach provides robust estimates of seasonal mobility changes in data-limited areas, and offers a foundational understanding of African mobility dynamics, which highlights the value of innovative modelling and novel sources to bridge data gaps for supporting regional planning and policy-making.

## INTRODUCTION

Understanding patterns in human mobility across spatial and temporal scales is essential for tackling critical challenges in development planning, public health and disaster response^1,2,3^. Human movement typically involves frequent short-distance trips punctuated by occasional long-distance travel, influenced by factors such as economic needs, family responsibilities, public holidays, seasonal climatic variations, and displacements resulting from natural disasters and political conflicts^4,3,5^. Despite its significance, accurately quantifying seasonal mobility at high spatial and temporal resolution remains a major challenge, particularly in low- and middle-income regions like Africa^6^. This challenge is compounded by the scarcity of comprehensive, longitudinal mobility datasets capable of capturing both domestic and cross-border dynamics.

Traditional methods, such as censuses, travel surveys, and traffic studies, have long been used to quantify population movements, especially migration^7,8^. However, these approaches often suffer from critical limitations: they are temporally infrequent, spatially coarse, and subject to recall bias. Moreover, they lack the flexibility to capture short-term or seasonal variations in mobility patterns^9^. Over a decade, call data records (CDRs) from mobile phones offer higher spatial and temporal granularity by collecting locational data through cell tower connections during billable events^10,11^. Yet, CDR data are constrained by their inability to track cross-border movements or continuous movements during a long-time gap of two billable events and are also heavily reliant on the density and distribution of cell towers of the same operators, which varies significantly across African countries^12^.

In recent years, passively collected location data from smartphones and internet check-in portable devices has emerged as a powerful tool to fill these gaps in quantifying mobility patterns at high spatial resolutions, spanning wide temporal periods, and across international borders ^13,14,8^. One such dataset is the Google Aggregated Mobility Research Dataset (GAMRD), which provides anonymised and aggregated mobility flows from users who have opted into the Location History^15,16^. This mobility dataset has been shown to be comparable with traditional data sources^17^. During the COVID-19 pandemic, GAMRD played a critical role in tracking mobility changes due to public health interventions, such as lockdowns and social distancing, and proved to be an invaluable resource for understanding shifts in population movement and assessing the effectiveness of non-pharmaceutical interventions in 2020–2022^18,19^. While GAMRD, like CDR data, may reflect certain sociodemographic biases, it remains a robust and timely source for analysing and revealing real-world mobility patterns, particularly in regions where traditional data are limited^15,20^.

However, significant knowledge gaps remain, especially in low- and middle-income countries across Africa^6^. Almost all African nations lack detailed seasonal baseline data on pre-pandemic mobility, which complicates efforts to understand how mobility patterns have evolved since the pandemic and how they may recover^16^. Additionally, the relationship between non-pandemic mobility changes and socioeconomic, climatic, and geographic factors remains unclear for many countries, especially in Africa.

This study aims to fill these critical gaps by leveraging GAMRD data to estimate seasonal human mobility across African countries with nearly complete data coverage in 25 countries, as well as partial data from 28 additional countries. Using a Bayesian spatiotemporal hierarchical modelling framework^5^, we measure pre-pandemic monthly mobility flows during 2018 and 2019 at both national and regional scales for countries throughout Africa. The model incorporates key geospatial covariates, including climate variables, socioeconomic indicators, and administrative boundaries, to explain the drivers of mobility. Through this approach, we generate robust, subnational estimates of seasonal mobility across Africa, offering a comprehensive, continent-wide perspective on human movement dynamics. The outputs of this study establish a mobility baseline and provide critical insights for informing policies in public health, economic resilience, and urban development in Africa.

## RESULTS

The Bayesian spatiotemporal regression models were constructed using the INLA package in R, following two independent modelling pathways: (1) 50 country-level models ( 25 outflow and 25 inflow models) to explore covariate effects on relative mobility changes for each country with nearly complete data coverage; and (2) 10 regional-level models (outflow and inflow models each for 5 African subregions) to estimate relative mobility changes for other African countries within the same region where GAMRD data were missing (See details in Methods). Two separate datasets were used across both modelling pathways, each for level 1 administrative units, one for outflow mobility (FR) models and the other for inflow mobility (TO) models. The relative mobility response was log_10_ transformed to comply with the Gaussian error assumption of the model; the resulting transformed mobility were checked via QQ-plot. Model parameter OLS estimates and corresponding Bayesian posterior means are tabulated along with their model performance metrics in the Supplementary Information 2.

**Figure 1:**
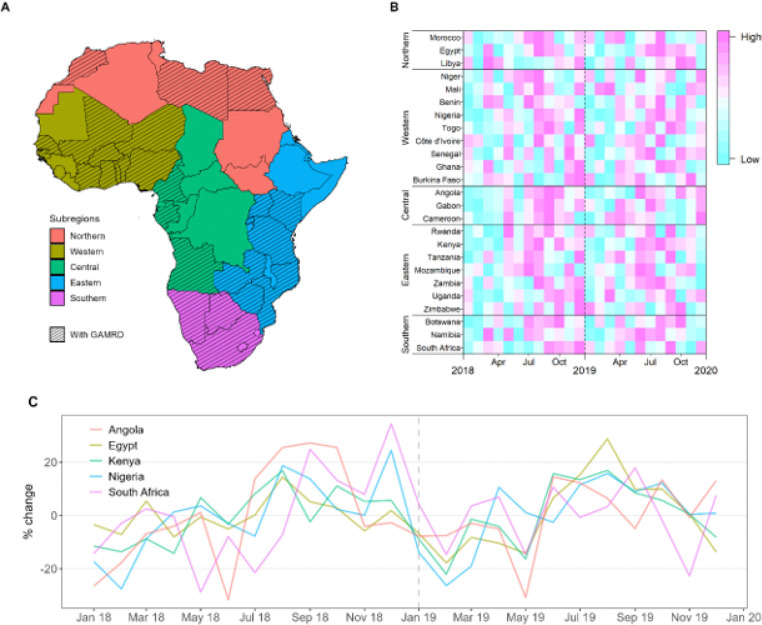
Data coverages and seasonal mobility patterns in Google Aggregated Mobility Research Dataset across Africa. **(A)** The 25 countries with available dataset are shown in black cross hatching. African countries are partitioned across five subregions. **(B)** The rank of monthly mobility of domestic and international travel. Months with higher volumes have a higher rank (from the lowest to the highest: 1–12) in each year. Each row in the heatmap represents a country grouped by UN African subregion. **(C)** Observed changes in monthly population flows among selected countries in each African subregions, as compared to the mean mobility level of each year.

UN defined subregions for Africa are shown in Figure 1A, where countries with nearly complete mobility coverage are mapped as a black cross hatch pattern. Also shown in Figure 1B are a heat map of relative seasonal mobility rankings for each year 2018 and 2019. Finally, a serial plot of mobility relative to annual mean mobility is shown in Figure 1C for a selection of countries, one from each UN subregion. Overall, countries show a relative high mobility from June to September.

### Country-level models

Common covariates across country-level models were mean accessibility, mean gross domestic product per capita, mean percent of infant mortality rate, mean percent of underweight children, percentage of urban extent, sum of urban extent, and mean percent of no primary education for males (Table 1). Country-level model with common covariates for simple main effects (second column) are listed separately from main effects in interaction terms (third column) of Table 1. ISO3 codes of countries in the second and third columns indicate which country-level model uses these common covariates.

Final spatiotemporal Bayesian outflow mobility (FR) and inflow mobility (TO) models for Kenya had no common two-way interaction terms between them indicating that direction of relative mobility flow is an important aspect to consider when analysing mobility for Kenya. Two common *simple* main effect terms between the two Kenya models (i.e. those not used in two-way interactions) were number of days for school holidays and maximum mean gross domestic product per capita. Details of parameter estimates for the Kenya models are given in the Supplementary Information 1 (see Tables S1, S2 for FR and TO models) with two-way interaction inference given in Table S3.

**Figure 2:**
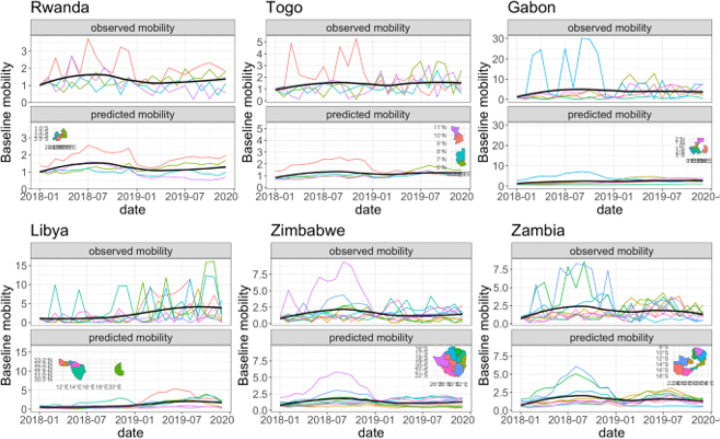
Series plots for observed and modelled relative mobility for selected countries with few county-level administrative units in 2018–2019. This series shows the mean behaviour of mobility trends across country-level administrative units (black line) while clearly illustrating that predicted behaviour tracks well with the observed values (coloured lines). Note predicted values for individual administrative units are mean trends modelled on observed mobility. Maps insets have incomplete administrative units as only those with mobility observations could be compared to modelled data. Administrative unit colours in maps matches the line colour for each plot.

A panel of series plots for select countries show observed and modelled data along with a map of provincial admin units in Figure 2. Observed relative mobility series is shown in the top half of each plot while the model predicted relative mobility series is on the bottom. The inset maps have admin unit colours that correspond with those of the observed and modelled series lines. Matabeleland North Province in east Zimbabwe (fuchsia line) has an observed spike in relative change in mobility in 2018 that does not occur at the same magnitude in 2019. The predicted relative change in mobility for Matabeleland North models the average (or smoothed) values of relative change in mobility over this same time with peak mobility values that correspond with those of the observed data. Other plots in the panel show similar behaviour for individual provinces within each country. The overall mean value of relative change in mobility (black line) across the country is shown for both observed and modelled data, each of which tracks similarly to one another over time.

### Regional-level models

Ten regional-level spatiotemporal Bayesian models were regressed for five regions where GAMRD data were available (Figure 1). Two datasets were used in regional-level modelling: one for the inflow model and one for outflow. Each model resulted in a unique set of covariates, but all had common random spatiotemporal indices of model specification (2). The model response for all models was a base-10 logarithm of a monthly mobility relative to the month of January in the same year. Provincial- and district-level mobility was predicted on a region-by-region basis for missing monthly mobility data within each of the five regions of Africa. Relative mobility was predicted for nearly 45% of provinces across Africa (8424 out of 18816) for which mobility data were either sparse or altogether missing over the two-year period of record. Missing mobility data counts by region for the outflow dataset as a percentage of regional total province counts were 72.6%, 60.1%, 37.4%, 28.2% and 26.9% for Central, Northern, Eastern, Western and Southern regions, respectively. Missing counts for inflow data were similar to those of outflow data.

**Figure 3:**
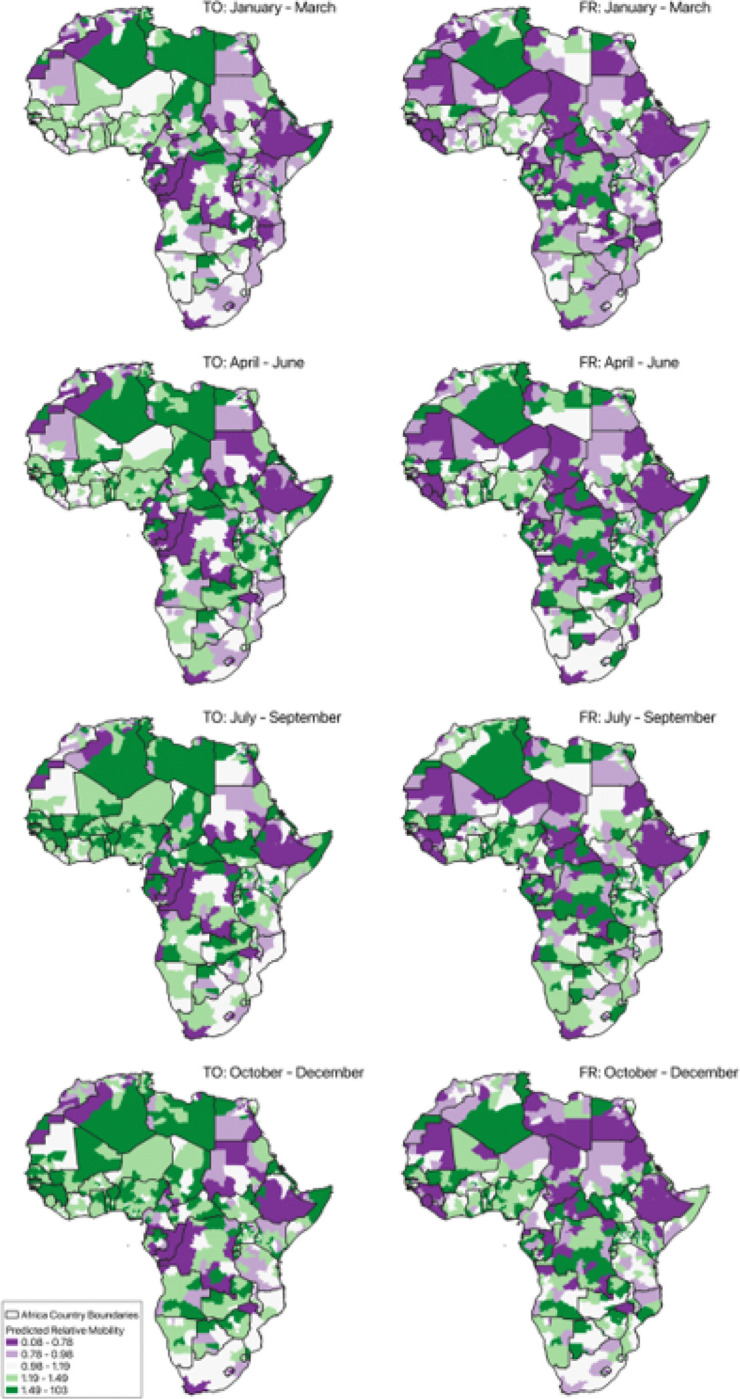
Predicted relative mobility for January to March, April to June, July to September, and October to December in 2018 across Africa (excluding islands). Predicted values are relative to mobility in January 2018, where values near unity indicates relatively little change from January 2018 (white), above unity have higher relative change (green), and values below unity have lower relative change (violet).

From the country-level analysis, we found from that 2018 had a higher variability than 2019; here we explore 2018 further at the regional-level. Continentwide predicted relative mobility maps for Africa are shown in Figure 3 consisting of four periods in 2018 (across rows). For inflow and outflow maps, predicted relative mobility values near unity (shown in white) indicate mobility counts for a given month were close to what they were in January 2018 (the baseline), values higher than unity (green) are higher mobility counts than in January 2018, and values lower than unity (violet) are lower mobility counts than in January 2018.

**Figure 4:**
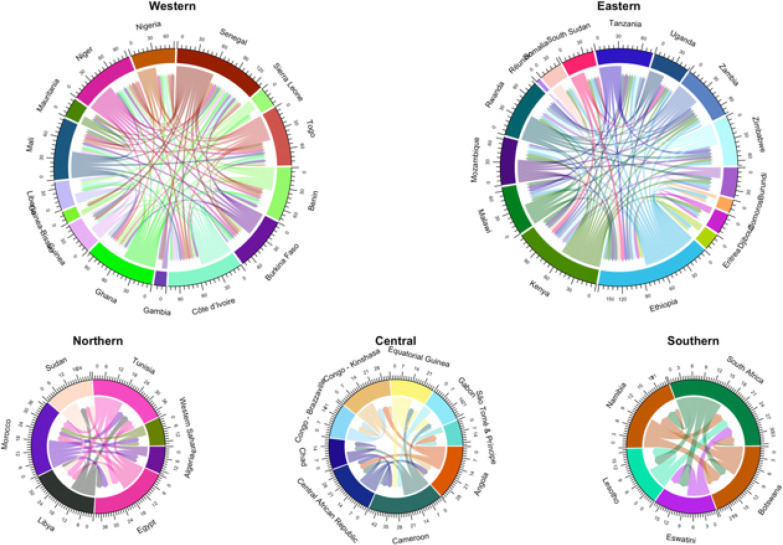
Direction of mobility, by magnitude, between countries within the five African regions measured in orders of magnitude (i.e. base-10 log transform of total relative mobility counts). Regional models were spatially restricted to mobility between countries within each region for 2018. Each regional chord diagram shows mobility occurs most often within country and between adjacent countries whereas mobility occurs least often between distant countries. However, there are a few acceptations where long distance mobility occurs between distant countries within a given region.

Spatial correlation is an important model component when predicting relative mobility in the five African regions. As most travel for any country within a region tends to be primarily within that country, or at least limited to adjacent countries, proximity of movement needs to be accounted for in the model. Model specification (2) has three spatial correlations considered in the regional model structure: , country-level indexing covering a regional extent; , provincial-level indexing at a country extent; and , provincial-level indexing over a regional extent (see Methods). Spatial correlation for regional outflow models had a mean range of 0.82 to 0.91, corresponding to most movements occurring at fairly close proximity. Indeed, chord diagrams of GAMRD data show directional movement between individual countries in 2018 within each African region (Figure 4). For the Southern region of Africa, Namibia has substantial internal movement and outflow movement to neighbouring countries of Botswana and South Africa to the east but no movement into Lesotho nor Eswatini. However, a few regions had significant movement beyond their adjacent countries. For example, in the Northern region, Egypt had outflows to its neighbouring countries of Sudan and Libya but also to distant countries of Morocco and Tunisia. Hence, the specified spatial correlation structure in regional models accommodates all mobility modelling scenarios. A single continentwide model was intractable as corelations between mobility response and spatiotemporal covariates were low enough as to be insignificant to the model.

**Figure 5:**
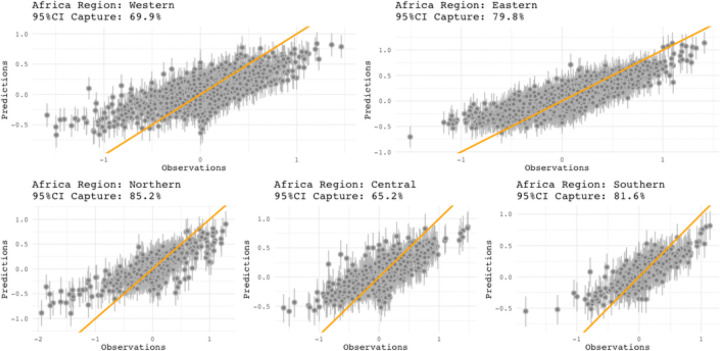
Calibration plots of predicted vs observed outflow (FR) relative mobility for five regions. The 95%Cl (credible interval) capture is noted for each region. Plots show mean prediction vs observations points (grey circles) each with a 95%Cl (grey vertical lines). The one-to-one line indicates a perfect fit (orange line). Calibration plots for inflow (TO) are similar but not shown.

Calibration plots to check model fit performance is shown in Figure 5. Fitting metrics for bias, imprecision, inaccuracy, and percent capture of a 95%Cl (credible interval) are also shown in Table 2. A binary variable to indicate domestic travel was included as a random and/or fixed term in the models, which were significant in models. However, a domestic travel indicator was eventually not included in the final prediction model since they are derived from GAMRD data and are not available for countries whose mobility is being predicted.

## DISCUSSION

This study presents a comprehensive spatiotemporal Bayesian analysis of human mobility across Africa using aggregated Google mobility dataset. By estimating and mapping relative mobility changes at subnational, national and regional levels, the findings address a critical data gap in regions where traditional mobility data are sparse or non-existent. Our research highlights the scale of mobility dynamics that exist, the predictability of models, and the benefits of bringing together multiple different forms of data to provide valuable insights for public health planning, infrastructure development, resource allocation, and economic resilience. These results are particularly timely as policymakers seek to understand how mobility patterns evolve post-pandemic and prepare for future disruptions.

A key finding of this study is the significant role that socioeconomic and environmental factors play in shaping mobility patterns. Covariates such as GDP per capita, underweight children, infant mortality, and environmental variables like stream runoff (excess precipitation that forms in rivers and lakes) and evapotranspiration (moisture that evaporates to the atmosphere and/or transpires via plants) were found to be significant predictors of mobility, particularly in outflow models. For example, in Kenya’s outflow model, the inclusion of these variables increased the adjusted coefficient of determination by 269% over the original case study model^5^, underscoring the importance of considering a broad range of factors in mobility analyses. This improvement aligns with previous research, which highlights the complex interplay between economic conditions and environmental factors in influencing migration and daily movement patterns^11,16^. For instance, studies on human mobility in West Africa have shown that harvest seasons and climatic factors such as rainfall and drought are critical determinants of seasonal migration, particularly in agrarian communities^21,22^. Our findings extend this understanding by demonstrating the added predictive power of socioeconomic indicators in conjunction with environmental variables.

We also revealed the importance of interaction effects between covariates, emphasizing that the contribution of one factor often depends on the levels of another. For example, the influence of GDP per capita on mobility outflow in Kenya was significantly moderated by urban extent, suggesting that wealthier areas with greater urban infrastructure experience different mobility dynamics compared to rural regions. This finding echoes global mobility studies that have shown how urbanization interacts with socioeconomic factors to shape seasonal movement patterns^12,4,23^, highlighting the need for tailored policy interventions based on localized conditions. Such insights are critical for policymakers seeking to manage urban growth or address rural-urban migration, as they underscore the necessity of context-specific strategies. Common two-way interactions for other countries are tabulated in the Supplementary Information 1 (Table S3), which includes inference of two-way interaction terms.

One notable contribution of this work is the use of regional-level models to estimate mobility patterns in countries where GAMRD data are incomplete or unavailable. By leveraging data from neighbouring countries within the same region, the models provide robust estimates of mobility changes in areas where direct measurement is not possible. This approach has important implications for regional planning and coordination, particularly in Africa, where both internal and cross-border mobility is common but often poorly documented^24,6^. Additionally, within the broader context of global mobility research, this study highlights the potential of passively collected data combined with spatiotemporal Bayesian modelling to fill significant data gaps. By incorporating diverse geospatial covariates, our models offer a practical solution for inferring mobility patterns in data-sparse regions, advancing understanding of regional mobility dynamics. These findings contribute to ongoing efforts to understand human movement in data-limited settings, providing a foundation for future research and policy development.

Several limitations should be acknowledged. First, the reliance on GAMRD data means that mobility patterns among populations without access to smartphones or who have not opted into Location History are not captured. This may result in underrepresentation of rural areas, where smartphone penetration is lower, and populations are more dispersed. The differential privacy algorithms used by Google to protect user anonymity further obscure fine-scale details, which may reduce the accuracy of mobility estimates in sparsely populated areas. Second, the representativeness of GAMRD data varies across countries and regions, as evidenced by incomplete coverage in some areas. These gaps highlight the need for additional data sources and methods to improve model coverage in future work. Combining GAMRD data with other sources, such as satellite imagery, transportation data, and travel surveys, could provide a more comprehensive understanding of mobility dynamics and their impacts^13,25,26^. Third, the models developed in this study are based on pre-pandemic data and are not designed to forecast mobility during periods of disruption, such as the COVID-19 pandemic. Mobility patterns during the pandemic were heavily influenced by lockdowns, travel restrictions, and other public health measures across the World^18,19,27,28^, which are not accounted for in the pre-pandemic models. Future research should focus on developing models that can accommodate sudden changes in mobility behaviour, such as those observed during disease outbreaks or natural disasters, e.g. flooding and tropical cyclones^3,14^. As data availability and modelling techniques continue to evolve, the ability to accurately monitor and predict mobility will play an increasingly important role in addressing the complex challenges facing African nations and the global community.

## METHODS

### Human mobility data

The Google Aggregated Mobility Research Dataset contains anonymized, aggregated mobility flows of users who have enabled the Location History setting on their smartphones, which is off by default. This is similar to the data used to show how busy certain types of places are in Google Maps–helping to identify when a local business tends to be the most crowded. The dataset aggregates flows of people from region to region, which is here further aggregated at county/ province and country levels. To produce this dataset, Google utilizes machine learning algorithms which automatically segment log data into semantic “trips”^29^. To provide strong privacy guarantees, all trips are anonymized and aggregated using a differentially private mechanism to aggregate flows over time^30,31^. This research is carried out on the resulting heavily aggregated and differentially private data. No individual user data was provided to researchers, only heavily aggregated flows of large populations were handled.

All anonymized trips are processed in aggregate to extract their origin and destination location and time. For example, if users travelled from location A to location B within time interval t, the corresponding cell (A,B,t) in the tensor would be n ± err, where err is Laplacian noise. The automated Laplace mechanism adds random noise drawn from a zero mean Laplace distribution and yields (ϵ,δ)-differential privacy guarantee of ϵ=0.66 and δ=2.1×10-29 per metric. Specifically, for each week W and each location pair (A,B), the number of unique users who took a trip from location A to location B during week W is calculated. To each of these metrics, Laplace noise from a zero-mean distribution of scale 1/ϵ is added. All metrics for which the noisy number of users is lower than 100 are removed, following the process described in^30^, and the rest is published. This yields that each published metric satisfies (ε,δ)-differential privacy with values defined above. The parameter ϵ controls the noise intensity in terms of its variance, while δ represents the deviation from pure ϵ-privacy. The closer they are to zero, the stronger the privacy guarantees.

The GAMRD dataset used in this study spans 2018 and 2019, with initial weekly data aggregated to a monthly timescale to align with the monthly covariates. Following the Kenya case study method^5^, we calculate the monthly mobility flow in Africa, relative to a January baseline within the same year, defined as the relative mobility change ratio

$$R_{c,a,m,y}=x_{c,a,m,y}/x_{c,a,Jan,y}$$

where x represents the monthly mobility flow (inward or outward) for each country c, level-1 administrative unit a (province or county), month m, and year y (2018 or 2019). This baseline standardizes mobility flows within each year, with $R_{c,a,Jan,y}=1$ and other values indicating higher $\left(R_{c,a,m,y}>1\right)$ or lower $\left(R_{c,a,m,y}<1\right)$ mobility relative to January baseline for any given location and year. Relative changes in human mobility are then modelled separately as either *outflow* or *inflow* across all country- and regional-level models.

### Study areas and modelling objectives

There are 54 countries on the African continent, each varying in human mobility patterns due to environmental, socioeconomic, and political differences. GAMRD data provides nearly complete spatial coverage at province and county levels for 25 African countries, while 28 additional countries have either sparse or incomplete coverage over the two-year study period. To contextualize our analysis, we follow the United Nation (UN) partitioning of the African continent into five subregions. Figure 1A shows the distribution of the 25 African countries with comprehensive data across the five subregions.

We purse two modelling pathways depending on objectives, both developed within a Bayesian framework that accounts for spatial and temporal autocorrelations:

#### Country-level models.

We first construct 50 country-specific models (25 outflow and 25 inflow), similar to those in the Kenya case study^5^. Here, our primary objective is to assess the impact of covariates, including significant two-way interactions, on relative changes in human mobility. These 50 models explore correlations specific to each country, providing a granular understanding of how different geospatial and sociodemographic covariates influence mobility within national boundaries.

#### Regional-level models.

We then develop 10 regional-level Bayesian models (5 outflow and 5 inflow) for each of the five African subregions. These models incorporate all available GAMRD data and aim to estimate incoming and outgoing relative mobility flows for countries with sparse or no data by leveraging patterns from neighbouring countries within the same region. Islands nations such as Madagascar are excluded due to the reliance of our spatiotemporal Bayesian models on spatially contiguous information from adjacent areas over multiple administrative levels, which is necessary for accurately capturing spatial autocorrelation.

### Geospatial covariates

We assembled a range of covariates for all African countries, exploring climatic, socio-economic, and demographic factors which could be linked to or potentially influence human mobility (Table 1). Contemporary raster data with a monthly temporal resolution was prioritised, to facilitate consistent geospatial and temporal aggregation, ensuring harmonisation across multiple data sources. Climate data was obtained for years matching that of GAMRD per capita, ensuring temporal coherence in the analysis. To reduce skewness in the distribution of certain covariates, logarithmic transformations were applied where necessary.

Raster data was extracted using Global Administrative Areas (GADM) shapefiles at administrative level 1 boundary (e.g., province or county). Zonal statistics, such as mean, median, minimum, maximum and sum of pixel values, were computed using ArcPy to capture relevant spatial characteristics for each administrative unit and monthly time step over the GAMRD period of record. Extraction ties statistics to a unique GID code which matches that of GAMRD data, thereby harmonising them and allowing comparison. To facilitate model inference readability, unit conversions and variable transformations were performed to standardise the range of values across covariates thus maintaining consistent numerical scales. This harmonisation minimises large disparities in parameter estimates, making the inference and comparison of model coefficients more straightforward. Details of each covariate, units, and transformations can be found in Table 3 and the Supplementary Information 1.

To prepare covariates for modelling, we examined them for potential issues such as skewness and multicollinearity. Highly skewed covariates underwent base-10 logarithmic transformation, facilitating interpretability by allowing changes to be expressed as orders of magnitude. To address multicollinearity in the model when including interaction terms, which is a challenge in statistical inference, all covariates were mean-centred for both country- and regional-level models. Additional scaling was applied for regional-level models to ensure comparability of estimates across different spatial scales (Table 3).

### Selecting model terms from available covariates

Before specifying Bayesian models for each country or region, it is important to identify model terms that yield the highest statistical significance since our main objective for country-level models is to infer coefficient correlations with relative changes in human mobility as part of an exploratory study. Stepwise regression is an efficient method for selecting an “optimal” set of model terms, particularly when two-way interactions are included because the number of terms to evaluate is substantial. Stepwise regression methods for Bayesian models, or Bayesian model averaging, do exist^32^; however, they are computationally intensive and slow when processing large datasets with complex hierarchical structures. Regardless of whether one uses Bayesian or ordinary least squares (OLS) regression, both model and error spaces are identical in each case.

Therefore, to optimise model specification from available covariates, we use OLS multiple linear regression as an initial step. This approach identifies statistically significant main effects and two-way interaction terms, which are subsequently incorporated into the Bayesian models. The use of OLS regression ensures computational efficiency in term selection while producing coefficient values and statistically significance results comparable to Bayesian models using the same data and model specifications. Moreover, OLS provides access to several model performance diagnostic tools such as predictive R^2^ and variance inflation factors (VIF)^33^, which are not readily available in Bayesian methods. These diagnostics help evaluate model fit, multicollinearity, and potential overfitting.

We performed OLS regression in the R programming platform^34^ using the MASS package^35^ stepAIC function in three stages: (1) a bidirectional stepwise regression of main effects using all covariates to obtain significant terms, (2) including potential two-way interaction terms using only significant main effects found in step 1, and (3) a backward stepwise regression with relaxed constraints on the exit criteria to eliminate marginally significant terms identified in step 2, thus resulting in a simplified model with higher overall estimate significance.

Once the stepwise regression stages are complete, models are manually checked for regression assumptions, fitting quality, multicollinearity, ill-conditioning, and overfitting (see performance metrics in the Supplementary Information 1). Models showing signs of either ill conditioning or overfitting are manually adjusted by dropping least significant terms and refitting the model until acceptable diagnostics are achieved. Finally, residual checks typically performed with any OLS regression complete the model evaluations^36^. The final set of OLS covariates found for each country or region is then used in the Bayesian models.

### Spatiotemporal Bayesian model specification

The dynamics of seasonal human movements evolves over both space and time, exhibiting distinct spatial and temporal autocorrelation patterns^5,12^. Spatially, mobility in a given area correlates more strongly with nearby areas than with distant ones. Temporally, mobility occurring at the current time step is usually influenced by movements that occurred in the previous time step but no sooner. These spatial and temporal autocorrelations, at the various spatiotemporal scales being considered, are inherent in human mobility data and must be accounted for in the statistical modelling framework^1^ To capture these dynamics, we employ the Integrated Nested Laplace Approximation (INLA), a Bayesian approximation method available through the R-INLA package^37^. INLA accommodates spatial and temporal autocorrelations using a suite of specialised autocorrelation functions. In our modelling framework, spatial dependencies are modelled using the Besag-York-Mollié (BYM) model^38^, for capturing the influence of neighbouring areas, while temporal dependencies are handled by the first-order autoregressive (AR1) model^39,37^, which considers the correlation between consecutive time steps. Both spatial and temporal autocorrelations plus their spatiotemporal interaction are considered in each model specification given by

1 $$\log\left(R_{c,a,m}\right)=A_{c,a}+B_{c,m}+C_{c,a,m}+X_{c,a,m}\beta_{c}+\epsilon_{c,a,m}\;for\;c=1,\ldots ,25,\;and$$

2 $$log\left(R_{r,a,m}\right)=\sum_{s=1}^{3}A_{r,a}^{\left(s\right)}+B_{r,m}+C_{r,a,m}+X_{\backslash varvecr,a,m}\beta_{r}+\epsilon_{r,a,m}\;for\;r=1,\ldots ,5,$$

where R is the mobility relative change ratio defined previously for each country c or UN subregion for Africa r, at level-1 administrative unit a, and month m of year y (2018 or 2019). A base-10 logarithmic transformation was used on the response to satisfy the Gaussian error assumption. Note the subscript for year y is dropped as data spanning both years are included in the model dataset. For model terms, $A_{c,a}$ and $A_{r,a}^{\left(s\right)}$ are the spatial autocorrelation functions based on the *bym2* model, $B_{c,m}$ and $B_{r,m}$ are the temporal autocorrelation functions based on the AR1 model, $C_{c,a,m}$ and $C_{r,a,m}$ are spatiotemporal autocorrelations that correspond to terms A and $B,\;X_{c,a,m}$ and $X_{r,a,m}$ are design matrices of spatiotemporal covariates including the intercept term, $\beta_{c}$ and $\beta_{r}$ are fixed model parameters for covariates to be estimated, and $\epsilon_{c,a,m}$ and $\epsilon_{r,a,m}$ are assumed Gaussian error terms for each specified model. In regional-level models, the three spatial correlation functions, $A_{r,a}^{\left(s\right)}$, reflect various combinations of spatial scales and areal extents: $A_{r,a}^{\left(1\right)}$ is a large-scale spatial correlation indexed at the country-level covering a regional extent, $A_{r,a}^{\left(2\right)}$ captures small-scale correlations at the subnational, administrative level 1 units with each country in a given region, and $A_{r,a}^{\left(3\right)}$ considers small-scale subnational spatial correlations across the entire regional extent. These multi-scale spatial terms ensure smoother region-to-region transition while preserving localised variations, thus reflecting the impact of local environmental and socioeconomic conditions on mobility. Models were sampled using INLA default priors for all random models and default Gaussian with mean zero and precision 0.001 for fixed model terms. A continentwide model was intractable due to a low covariate correlation with the response for a single model, necessitating separate country-level and regional models to maintain statistical robustness of accurate estimation and interpretation of mobility patterns across Africa.

Calibration plots are used to assess Bayesian model performance by plotting model predicted posterior mean relative mobility values against the observed relative mobility values. Credible intervals are the inner 95% of sampled points for each sampled mobility distribution corresponding to an observed mobility value. Capture occurs where the one-to-one predicted-to-observed line are contained within the 95% credible intervals (corresponding to a 5% significance). Fitting metrics for bias, imprecision, inaccuracy, and percent capture of a 95%CI (credible interval) will be included in the Bayesian model performance metrics. Model residuals are the difference between observed values and predicted values given by the model, which are used in assessing model fit. Bias is the mean of all residuals, where bias values closer to zero indicate better results. Imprecision is the standard deviation of all residual values, which indicates the amount of data scatter of observed data around the regressed model. Inaccuracy is the mean absolute value of model residuals, which is the mean distance that observed values are from the regressed model as either an overestimate or an underestimate.

## Figures and Tables

**Table 1.** Common significant (at *p* < .05 level) simple main effect covariate terms listed in the first column for outflow (FR) and inflow (TO) mobility models for 22 out of 25 African countries. Country models specified with covariate as main effect terms are listed in the second column while country models specified with covariates included as interaction terms are listed in the third column.

Common covariate	Country models with main effects only	Country models with interaction terms

mean_ACCS	CMR, MLI, NGA, RWA, SEN, ZWE	AGO, BFA, GHA
mean_GDPC	AGO, BEN, EGY, NAM, ZMB	BWA, CIV, CMR, KEN, TZA, UGA
mean_INFM	BEN, MOZ, NAM, NER, SEN, ZMB, ZWE	BFA, NGA, UGA
mean_UWCH	AGO, NGA, SEN, TZA, ZWE	BEN, BWA, CIV, CMR, UGA
prop_URBN	GHA, RWA, TZA, UGA, ZMB, ZWE	CIV, CMR, MAR
sum_URBN	AGO, BEN, CIV, EGY, GAB, MOZ, NGA, TZA	BFA, CMR, KEN, UGA
mean_NPRM	CIV, GAB, MAR, MOZ, NER, ZMB, ZWE	--

**Table 2.** Fitting metrics for regional inflow and outflow relative mobility models.

Fitting metrics for regional relative mobility models for Africa					
Model	Region	Bias	Imprecision	Inaccuracy	Captures 95%CI
Inflow	Central	−2.17 × 10^−3^	0.296	0.226	62.2
	Eastern	−3.50 × 10^−8^	0.186	0.142	73.8
	Northern	−3.64 × 10^−8^	0.225	0.143	86
	Southern	−2.37 × 10^−8^	0.23	0.164	62.2
	Western	−4.00 × 10^−8^	0.192	0.141	77.3
Outflow	Central	−1.39 × 10^−3^	0.284	0.222	65.2
	Eastern	−3.44 × 10^−8^	0.179	0.135	79.8
	Northern	−3.32 × 10^−8^	0.233	0.144	85.2
	Southern	−2.32 × 10^−8^	0.184	0.129	81.6
	Western	−3.73 × 10^−8^	0.219	0.158	69.9

**Table 3.** Potential covariates used in country- and regional-level models. The CODE column lists the variable names used in models, which facilitates readability of model outputs. Covariates are described along with their respective units and any applied variable transformations. The intervals in the last column are the minimum and maximum of mean covariate values for all level-1 administrative units across Africa. Base-10 logarithmic transformations were used where necessary to ensure that covariate inference is described in orders of magnitude for enhanced interpretability. For data source and processing details, see the Supplementary Information 1.

CODE	Covariate	Units and Transformations (mean, sum)	Range of Means
DWPT	2m Dewpoint Temperature	C	[−10.43, 25.21]
TEMP	2m Surface Temperature	C	[2.71, 37.49]
EVAP	Evaporation	mm/day, sums are log10(mm/day + 1)	[0.02, 6.46]
LAIH	LAI High Vegetation	m2/m2	[0, 5.98]
LAIL	LAI Low Vegetation	m2/m2	[0, 4.05]
PREC	Precipitation	mm/day, sums are log10(mm/day + 1)	[0, 37.37]
SKRC	Skin Reservoir Content	mm/day, sums are log10(mm/day + 1)	[0, 0.55]
PRES	Surface Pressure	kPa	[74.72, 102.12]
ROFF	Total Runoff	mm/day, sums are log10(mm/day + 1)	[0, 32.07]
WINU	Wind U (Eastward)	m/s	[−5.53, 6.82]
WINV	Wind V (Northward)	m/s	[−7.45, 8.33]
ACCS	Accessibility	log10(minutes)	[−1.36, 3.58]
INFM	Infant Mortality Rate	percent	[1.04, 20.23]
NPRF	No Primary Education, Female	percent	[1.39, 49.74]
NPRM	No Primary Education, Male	percent	[1.11, 49.08]
SECF	Secondary Education, Female	percent	[0.13, 61.1]
SECM	Secondary Education, Male	percent	[1.16, 64.22]
UWCH	Underweight Children	percent	[1.13, 54.7]
GDPC	Mean GDP Per Capita	log10(USD per capita)	[2.88, 6.59]
NTLS	VIIRS Night-time Lights	log10(nW/cm2/sr + 1)	[−0.01, 1.7]
URBN	Urban Extent	percent, sums are log10(count + 1)	[0, 99.74]
HOLS	Public Holidays	days	[0, 5]
HSCH	School Holidays	days	[0, 31]

## Data Availability

Modelling code in the R statistical platform and output mobility estimates are available on the GitHub. The covariate data used in this study are either openly available or under the licensing agreements, which can be made available upon request to the corresponding authors. The Google COVID-19 Aggregated Mobility Research Dataset used for this study is available with permission from Google LLC. Ethical clearance for collecting and using secondary data in this study was granted by the institutional review board of the University of Southampton (No. 48002 and No. 87924). All data were supplied and analysed in an anonymous format, without access to personal identifying information.
